# Laser-induced breakdown spectroscopy coupled with machine learning as a tool for olive oil authenticity and geographic discrimination

**DOI:** 10.1038/s41598-021-84941-z

**Published:** 2021-03-08

**Authors:** Nikolaos Gyftokostas, Dimitrios Stefas, Vasileios Kokkinos, Christos Bouras, Stelios Couris

**Affiliations:** 1 Department of Physics, University of Patras, 26504 Patras, Greece; 2 Institute of Chemical Engineering Sciences (ICE-HT), Foundation for Research and Technology-Hellas (FORTH), Patras, Greece; 3 Department of Computer Engineering & Informatics University of Patras, 26504 Patras, Greece

**Keywords:** Optical spectroscopy, Laser-produced plasmas, Characterization and analytical techniques

## Abstract

Olive oil is a basic element of the Mediterranean diet and a key product for the economies of the Mediterranean countries. Thus, there is an added incentive in the olive oil business for fraud through practices like adulteration and mislabeling. In the present work, Laser Induced Breakdown Spectroscopy (LIBS) assisted by machine learning is used for the classification of 139 virgin olive oils in terms of their geographical origin. The LIBS spectra of these olive oil samples were used to train different machine learning algorithms, namely LDA, ERTC, RFC, XGBoost, and to assess their classification performance. In addition, the variable importance of the spectral features was calculated, for the identification of the most important ones for the classification performance and to reduce their number for the algorithmic training. The algorithmic training was evaluated and tested by means of classification reports, confusion matrices and by external validation procedure as well. The present results demonstrate that machine learning aided LIBS can be a powerful and efficient tool for the rapid authentication of the geographic origin of virgin olive oil.

## Introduction

The Mediterranean region countries are the most important olive oil producers, producing almost 69% of the worldwide olive oil production today^1^, as their climatic conditions favor the cultivation of olive trees, while being responsible for the main characteristics of olive oil (e.g. its fatty acid composition, color, flavor, etc.). Olive oil is considered as a key element of human diet because of its high nutritional value, while its consumption is related to longevity and healthier living being widely recommended against heart and circulatory diseases^2^. All these resulted to a steadily increasing olive oil demand and value. Inevitably, this situation makes olive oil prone to adulteration and to other illegal and dangerous for the health activities^3^. Therefore, the necessity to ensure the quality of olive oil becomes of high importance. Adding cheaper and/or lower quality olive oil (as e.g., not fulfilling the PDO/PGI characterizations) is among the most common adulteration practices and besides the financial aspects, it raises ethical and (sometimes) health problem issues as well. These fraud attempts have established food safety as a critical issue in public opinion, research, professional management, and government regulations worldwide^4^. In that view, various international organizations, such as the Codex Alimentarius and the International Olive Council (IOC) have developed regulations on food safety and authenticity to confront these issues^5,6^. In Europe, these regulations are established not only for human health protection but also to protect consumers’ interests and rights. Consumers are entitled to know exactly the content of the product they are purchasing, as well as its origin^7^. Consequently, accurate and reliable labelling is crucial to inform consumers correctly; thus, olive oils (and foodstuff, in general) must be labelled with a full description of the product, i.e., list of ingredients, designation of origin, etc. In that view, the European Union (EU) has established frameworks acting as guidelines to the consumers such as, the Protected Designation of Origin (PDO) and the Protected Geographical Indication (PGI) which characterize the different olive oils and refer to the properties and quality of olive oil derived from its region of origin. The PDO/PGI characterizations of olive oil are criterions for the authenticity and quality, while act as a guarantee of the brand name of an olive oil^4,8,9^.

The above-mentioned issues have mobilized many scientists, from different research areas, to develop techniques for olive oil characterization (PDO/PGI)^10,11^, detection of adulteration^12–14^, etc. For the geographical discrimination of olive oil several techniques have been proposed, such as near-infrared reflectance spectroscopy (NIR)^15^, spectral nephelometry^16^, chromatographic and spectroscopic techniques including Inductively Coupled Plasma Atomic Emission Spectroscopy (ICP-AES) and stable isotope ratios measurements^17^, and visible and near-infrared spectroscopy^18^. Other techniques, which are much more accurate and widely used are the high-performance liquid chromatography (HPLC)^19^ and the Nuclear Magnetic Resonance spectroscopy (NMR)^20^. Moreover, extensive research has been conducted on the use of volatile compounds for the geographic discrimination of olive oils^21–23^. They exhibit high sensitivity, however they require expensive equipment and highly qualified personnel, while they are time-consuming due to the sample preparation procedures required. Therefore, a rapid, lower cost, reliable and accurate enough method can be of great importance and utility for olive oil quality control and analysis needs.

Laser-induced breakdown spectroscopy (LIBS) fulfils the above criteria. LIBS is a laser based optical spectroscopy technique, usually employed for the elemental composition of different materials^24^. LIBS can detect the atomic and molecular emissions arising from the elements and (usually) small molecular species, present or produced in the plasma resulting during the interaction of intense laser beams with a sample^24,25^. Very recently, LIBS has been proposed as a tool for food analysis purposes^26^ as well. In more details, a sample is exposed to a focused laser beam, absorbing the laser photons, heated and becoming partially excited and ionized, leading to the formation of a micro-plasma (plasma plume). The radiation emitted from the plasma contains information about the elemental composition of the sample, while emissions from molecular species of the sample or fragments of molecules generated within the plasma plume can be also present and emit characteristic emissions. These emissions can be collected and spectrally analyzed, thus providing a kind of fingerprint of the sample. The two major advantages of LIBS technique that make it very attractive and popular are that all states of matter (i.e., solid, liquid or gaseous) can be analyzed, independently if they are conductive or dielectric, while no sample preparation is required. These advantages, together with LIBS capability for in-situ or remote real time operation make LIBS highly attractive and have led LIBS to gain high popularity in the research community. Thus, over the years, LIBS has been applied in a variety of research areas with great success, including recycling of metallic and/or plastic materials^27,28^, metallurgy^29,30^, art conservation^31^ and combustion diagnostics^32^ to mention only some of them. However, it is only recently that LIBS has been proposed for food research related applications, coinciding with the rapidly increasing recognition of the effectiveness of machine learning approaches for analytical purposes and spectroscopic analysis^33–36^. The main reason for this delayed application of LIBS is due to the fact that the LIBS spectra of organic matter (e.g., foodstuff, olive oil, etc.) are very similar as these organic materials have similar elemental compositions, and are very difficult to be analyzed by means of more conventional methods (as e.g. calibration curves, PLS approaches, etc.). Instead, the machine learning based search for patterns formation and/or correlations within the LIBS spectral data seems to be a highly promising and less explored research direction so far. In fact, the combination of LIBS with machine learning algorithms seems to open new possibilities in the fields of laser-based analysis of materials, food science and safety and food quality control. Although this field of applications is very new, it has resulted to very promising results so far, while it leaves a lot of space for future research, development, and improvement^36,37^. In the present work, LIBS assisted by machine learning algorithms is used for the first time, to the best of our knowledge, to discriminate several extra virgin (EVOO) and virgin (VOO) olive oil samples regarding their geographical origin. The great majority of the studied olive oil samples were characterized as extra virgin olives (EVOOs) while few were characterized as virgin olive oils (VOOs). Special emphasis is given in the determination of the most important spectral features in terms of their contribution in the discrimination procedure, aiming to connect the different spectral features exhibited in the plasma emission with the degree of successful classification by machine learning. The above issues are studied here aiming to show and validate the high potential of machine learning aided LIBS for olive oil classification in terms of its geographical origins^38–40^.

## Results and discussion

### Olive oil LIBS spectra

Some representative LIBS olive oil spectra are shown in Fig. S1 (see e.g. Supplementary Material), where the most important spectral features are clearly observed. The assignment of the spectral lines was based on the National Institute of Standards and Technology (NIST) Atomic Spectra Database (ASD) and are also confirmed by other works and from our unpublished data^33,34,41^. As can been seen, both atomic and molecular origin emissions are apparent, resulting from the atomization and/or fragmentation of the molecular constituents of olive oil. Thus, the atomic hydrogen emissions at 656.3 nm and 486.1 nm (i.e., the Balmer H_α_ and H_β_ lines respectively), the oxygen emissions O I at 777.2, 777.4 and 777.5 nm spectral lines (centered at about 777 nm), and the O I at 615.7, 715.7 and 926.4 nm emission lines are also observed. Furthermore, the two triplets of nitrogen emissions at N I (at 742.4, 744.2, 746.8 nm and 818.8, 821.6, 824.2 nm respectively) are clearly observable together with the carbon lines: C I at 247.9, 795.2, 833.5 nm, 906.2 and 940.6 nm appear among the more intense spectral features (see also Supplementary Material, Table S1). Moreover, the vibrational progressions of the C_2_ Swan band system (around 500 nm), the CN violet band system (around 388 nm) are also clearly observable and among the most prominent spectral features of the olive oil plasma emission. These molecular bands are generally observed in the LIBS spectra of organic matter^42,43^. Table S1 at the supplementary material section lists the observed spectral features appearing in the LIBS spectra.

From the inspection of the LIBS spectra of the olive oil samples it becomes evident that they all exhibit the same emission lines and very similar spectral patterns, with slight differences of their relative intensities. Consequently, any discrimination/classification attempt of the olive oil samples by simple inspection of the LIBS spectra seems rather very difficult if not impossible. In that view, machine learning algorithms seem to be a suitable tool to capture the subtle changes of the spectral features’ relative intensities and identify any eventually spectral patterns which are not observable by simple LIBS spectra inspection, therefore offering a possibility for a classification of LIBS olive oil spectroscopic data.

### Classification results from the linear discriminant analysis (LDA)

**Figure 1:**
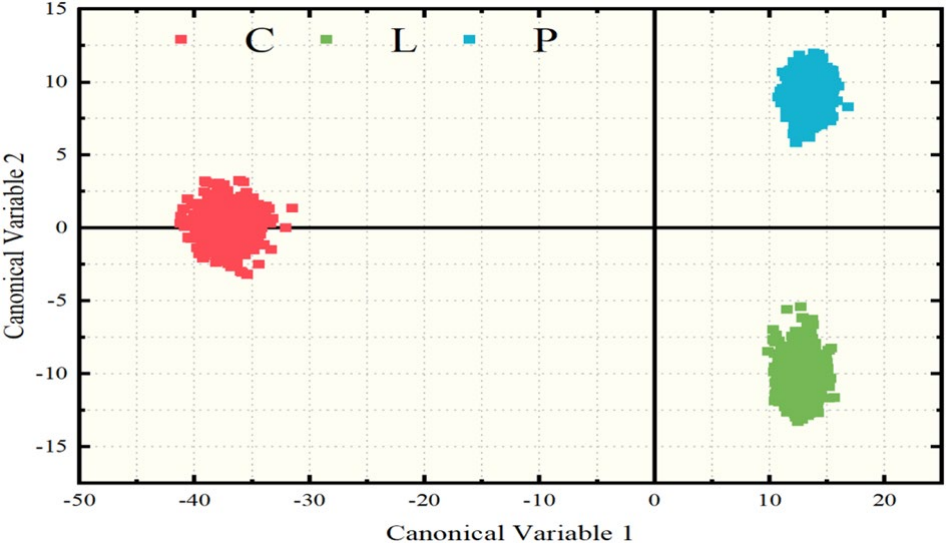
LDA canonical variables plot for the olive oil samples originating from different geographical regions. Red data points correspond to samples from Crete (C), green and blue data points correspond to samples from Lesvos (L) and Peloponnese (P) respectively.

At first, the LDA algorithm was applied to the LIBS spectroscopic data. The algorithm was trained using the maximum number of canonical variables allowed, which is equal to the number of classes (i.e., 3) reduced by one; thus, the maximum number of canonical variables used were 2. In Fig. 1, the corresponding LDA scatter plot of the LIBS spectroscopic data is presented. As shown, the formation of three distinct clusters is clearly observable. The red, blue, and green colored clusters correspond to samples originating from Crete (C), Lesvos (L) and Peloponnese (P), respectively. The estimated accuracy of the algorithm attained practically the value of (100.0 ± 0.0) %, as it was expected, from the observation of the LDA scatter plot of the samples of the three regions, which indicates that the three regions are remarkably distinguishable from one another. A more detailed picture for the internal evaluation of the applied algorithm is provided by the corresponding classification report and confusion matrix which are presented in Table 1a and b, respectively. The precision, the recall, the f1-scores and the support parameters are given in Table 1a. Among these parameters, the f1-score parameter is selected for the needs of the present discussion, as it contains information from both the precision and the recall parameters. The f1-scores are valued between 0 and 1, usually indicating successful classification of a sample when its value is larger than 0.5 or unsuccessful classification for values lower or equal than 0.5. As can been seen from Table 1a, all f1-scores attained the value of 1 (italic), suggesting that all samples were predicted correctly. This result is further confirmed from the confusion matrix shown in Table 1b, where the correct predictions are presented, corresponding to the diagonal elements of the matrix (italic). Furthermore, the macro average score was found to get the value of 1 (i.e., 100%), being the same with the obtained accuracy from the algorithmic training, suggesting a well-trained model. To further evaluate the LDA predictive model, an external validation procedure was performed as well. For this evaluation, 4 samples from each region (i.e., 12 samples in total) were randomly removed from the training procedure and they were used only for testing purposes. Then, the remaining 127 samples were used for the algorithmic training. The corresponding confusion matrix is presented in Table 1c. The predicting accuracy was found to attain a value of 100%, all the samples being correctly classified according to their geographical origin. These results are summarized in Table 1c.

**Table 1:** (a) Classification report, (b) confusion matrix for internal validation, and (c) confusion matrix for external validation resulting from the LDA algorithm.

Linear Discriminant Analysis (LDA)									
a) Classification report									
	Precision	Recall	f1-score	Support					
C	1	1	*1*	105					
L	1	1	*1*	139					
P	1	1	*1*	173					
Accuracy	1	1	*1*	1					
Macro avg	1	1	*1*	417					

(b) Confusion matrix (internal validation)					(c) Confusion matrix (external validation)				
		Predicted class					Predicted class		
		C	L	P			C	L	P
Actual class	C	*105*	0	0	Actual class	C	*120*	0	0
	L	0	*139*	0		L	0	*120*	0
	P	0	0	*173*		P	0	0	*120*

### Classification results using the Extremely Randomized Trees Classifier (ERTC) algorithm

In the next, the LIBS spectra were used for the training of the ERTC algorithm. The accuracy of the corresponding predictive model attained a value of (100.0 ± 0.1) %, suggesting a very well-trained and robust model. For a more detailed insight, the classification reports and confusion matrix were constructed and are presented in Table 2a and 2b, respectively. As can been seen, the f1-scores got the value of 1 for all olive oil samples (see e.g. Table 2a), denoting that all the samples were predicted correctly (see e.g. Table 2b). For further evaluation of this predictive model, an external validation procedure was performed, similar to that described previously for the case of the LDA algorithm. The accuracy of the new trained model remained unchanged after the removal of the 12 samples from the total number of samples. The obtained results are presented in the form of confusion matrix in Table 2c. The accuracy of the predicted samples was found to attain a value of 100%, predicting perfectly all the samples, indicating that the ERTC predictive model is highly effective and robust as well.

**Table 2:** (a) Classification report, (b) confusion matrix for internal validation, and (c) confusion matrix for external validation resulting from the ERTC algorithm.

Extremely randomized trees classifier (ERTC)									
(a) Classification report									
	Precision	Recall	f1-score	Support					
C	1	1	*1*	105					
L	1	1	*1*	139					
P	1	1	*1*	173					
Accuracy	1	1	*1*	1					
Macro avg	1	1	*1*	417					

(b) Confusion matrix (internal validation)					(c) Confusion matrix (external validation)				
		Predicted class					Predicted class		
		C	L	P			C	L	P
Actual class	C	*105*	0	0	Actual class	C	*120*	0	0
	L	0	*139*	0		L	0	*120*	0
	P	0	0	*173*		P	0	0	*120*

**Figure 2:**
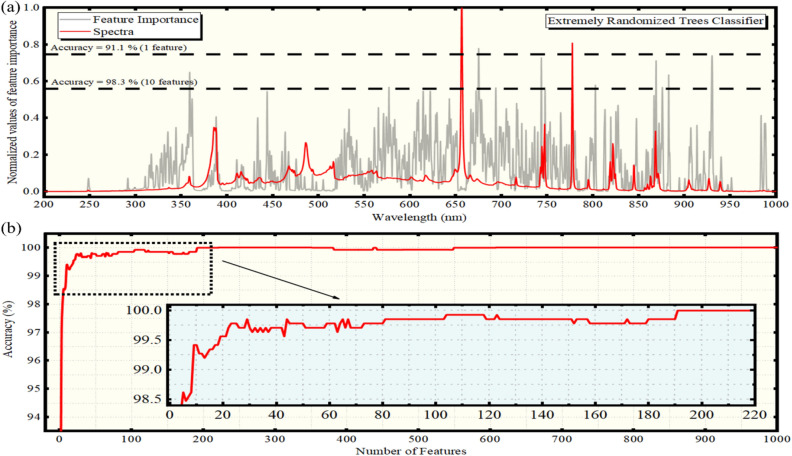
(**a**) Feature importances (gray line) versus wavelength resulting from the ERTC algorithm. An experimental LIBS spectrum (red line) is shown for comparison. The horizontal dashed lines indicate the number of features threshold and the corresponding computed accuracies. (**b**) Prediction accuracy using the ERTC algorithm versus the number of features.

Next, the importance of the different spectral features appearing in the LIBS spectra was obtained using the ERTC algorithm. The obtained results are shown in Fig. 2a. The red line corresponds to a LIBS spectrum of an olive oil, while the grey colored lines indicate the importance of the different spectral features calculated by the algorithm. The accuracy of the algorithm per feature is also presented in Fig. 2b, indicated by the horizontal dashed lines. As shown, by considering only the N I 672.6 nm line, i.e., only one spectral feature, an estimated accuracy of 91.1% was obtained. The importance of this spectral feature is indicated by the grey colored lines in Fig. 2a. Including more spectral features for the algorithm training (or equivalently by reducing the threshold of variable importance), the accuracy of the algorithm was found increasing. As an example, after including 10 spectral features, the algorithm attained an accuracy as high as 98.3% (see e.g. Fig. 2a).

Among the most important spectral features are the CN (Δν =  + 1) band, the C I 795.2 nm and 843.1 nm atomic emissions, the two N I triplets centered around 744 and 820 nm, and multiple N I spectral lines at about 865 nm and the O I 926.4 nm line. The contribution of the continuum background was found to be rather weak for the discrimination procedure since, only with the aforementioned emissions the algorithm attained quite high accuracy. Similar behavior was observed for the H_γ_ 434.1 nm, multiple C I spectral lines (i.e., 247.9 nm, 906.2 nm, and 940.6 nm), the CN (Δν = 0, + 1) band and the O I 615.7 nm and 715.7 nm lines, together with the O I triplet located around 777 nm. All these spectral features were found to have relatively weak contribution to the accuracy of the algorithm.

It is useful to remind at this point, that the main olive oil constituents (corresponding to 98–98.5%) are the oleic acid (C_18_H_34_O_2_), the linoleic acid (C_18_H_32_O_2_), the palmitic acid (C_16_H_32_O_2_) and glycerol (C_3_H_8_O_3_), i.e. molecules consisting from C, O and H. Therefore, at first glance, it is reasonable to expect that emissions arising from these atoms to contribute the most in the discrimination/classification procedure. Interestingly, except C and O atomic lines emissions, which contribute significantly, the H atom emissions were found to have a rather weak influence on the performance of the algorithm. In contrast, the N I emissions were found to have significant contribution. It is reminded that nitrogen atoms are meet in chlorophyll, the natural pigment accounting for the greenish color of the olive oil. However, as N_2_ exists in air and the present experiments are performed under ambient conditions, some N I emissions, may result from the fragmentation of the atmospheric N_2_ under plasma conditions. Thus, in principle they cannot be excluded. To assess their influence/contribution on the observed N I emissions of the LIBS spectra of olive oil, two different experiments were performed. In the first type of experiment, some argon flow was constantly flashed on the surface of the olive oil sample, to reduce/remove the air above the samples. In the second experiment, the sample was placed in an air-tight cell, under Ar atmosphere. In both cases, the N I emissions were found to be weakly affected compared to those of the experiments carried out under air ambient atmosphere (see i.e., Fig. S2 in Supplementary Material).

### Classification results using the random forest classifier (RFC) algorithm

Next, the Random Forest Classifier (RFC) algorithm was used for the classification of the LIBS spectroscopic data. The corresponding classification report and the confusion matrix of the RFC predictive model are presented in Table 3a and b, respectively. The attained accuracy was found to be as high as (99.8 ± 0.3) %. As the estimated accuracy reached almost 100%, all the f1-scores of the classification report (see Table 3a) were found to be equal to 1 (i.e., 100%). Similarly, the value of the macro average was found to be equal to one. As expected from these results, all the predictions, shown in the confusion matrix presented in Table 3b, were all found to be correctly predicted. Next, an external validation for the RFC predictive model was performed. The corresponding confusion matrix is presented in Table 3c. The trained RFC algorithm attained the same accuracy, with the prediction accuracy of the new samples attained a value of 100% (see Table 3c). These results suggest a very successful operation of the RFC predictive model for the classification of olive oils according to their geographic origin.

**Table 3:** (a) Classification report, (b) confusion matrix for internal validation, and (c) confusion matrix for external validation resulting from the RFC algorithm.

Random Forest Classifier									
(a) Classification report									
	Precision	Recall	f1-score	Support					
C	1	1	*1*	105					
L	1	1	*1*	139					
P	1	1	*1*	173					
Accuracy	1	1	*1*	1					
Macro avg	1	1	*1*	417					

(b) Confusion matrix (internal validation)					(c) Confusion matrix (external validation)				
		Predicted class					Predicted class		
		C	L	P			C	L	P
Actual class	C	*105*	0	0	Actual class	C	*120*	0	0
	L	0	*139*	0		L	0	*120*	0
	P	0	0	*173*		P	0	0	*120*

**Figure 3:**
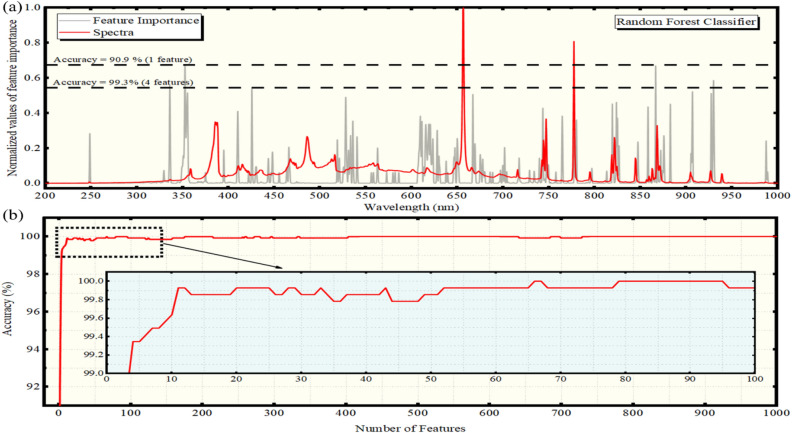
(**a**) Feature importances (gray line) versus wavelength resulting from the RFC algorithm. An experimental LIBS spectrum (red line) is shown for comparison. The horizontal dashed lines indicate the number of features threshold and the corresponding computed accuracies. (**b**) Prediction accuracy using the RFC algorithm versus the number of features.

Furthermore, the contribution of the most prominent spectral features of the LIBS spectra for the training of the algorithm was investigated. The determined feature importance of the different spectral features are shown in Fig. 3a. The estimated accuracy of the predictive model per feature is shown in Fig. 3b. As can been seen, by using only the CN (Δν = − 1) molecular band emission for the algorithmic training, an accuracy of 90.9% was achieved. The addition of the NH band emission at 336.3 nm^44,45^, N I 868.3 nm and O I 926.4 nm spectral lines for the algorithm’s training, i.e. 4 spectral features in total, resulted to an accuracy of the predictive model of 99.3%. Other emissions (as e.g., those of: C I 247.9 nm, C I 426.9 nm and C I 906.2 nm, O I 615.7 nm, O I 672.6 nm, O I 777 nm, N I 694.6, N I 698.1, N I 744.2, N I 746.8 nm) were found to have much weaker contribution. It is interesting to add here, that the importance of the continuum on the algorithmic training was found to be reduced significantly compared to the importance found in the case of the ERTC algorithm. Thus, comparatively, it seems, that in the case of the RFC algorithm the spectral lines have a more important contribution than in the ERTC algorithm. An explanation could be that ERTC is more sensitive to noisy features (see Data Analysis Section) compared to the other algorithms.

### Classification results using the extreme gradient boosting classifier (XGBoost) algorithm

Last, the XGBoost algorithm was used for the construction of a predictive model for the classification of the LIBS spectra of the different olive oils and for the assessment of the corresponding feature importances, as well. The accuracy attained by the predictive model was as high as (99.8 ± 0.2)%. For a more detailed view of the algorithmic training, the classification report and the confusion matrix of the model were constructed and are presented in Table 4a and b, respectively. The values of the f1-scores were equal to 1 (i.e., 100%) and the accuracy along with the macro average score were also 1 (see e.g. Table 4a), suggesting a well-trained predictive model. In addition, all the tested samples following internal validation were correctly classified, as indicated by the corresponding confusion matrix shown in Table 4b. To ensure the robustness of the algorithm and that it has been fitted and trained successfully, an external validation procedure was also performed. The results of this external validation are shown in Table 4c. As can be seen, the unknown samples were all predicted correctly, thus confirming the robustness of the constructed predictive model.

**Table 4:** (a) Classification report, (b) confusion matrix for internal validation, and (c) confusion matrix for external validation resulting from the XGBoost algorithm.

eXtreme Gradient Boosting Classifier									
(a) Classification report									
	Precision	Recall	f1-score	Support					
C	1	1	*1*	105					
L	1	1	*1*	139					
P	1	1	*1*	173					
Accuracy	1	1	*1*	1					
Macro avg	1	1	*1*	417					

(b) Confusion matrix (internal validation)					(c) Confusion matrix (external validation)				
		Predicted class					Predicted class		
		C	L	P			C	L	P
Actual class	C	*105*	0	0	Actual class	C	*120*	0	0
	L	0	*139*	0		L	0	*120*	0
	P	0	0	*173*		P	0	0	*120*

**Figure 4:**
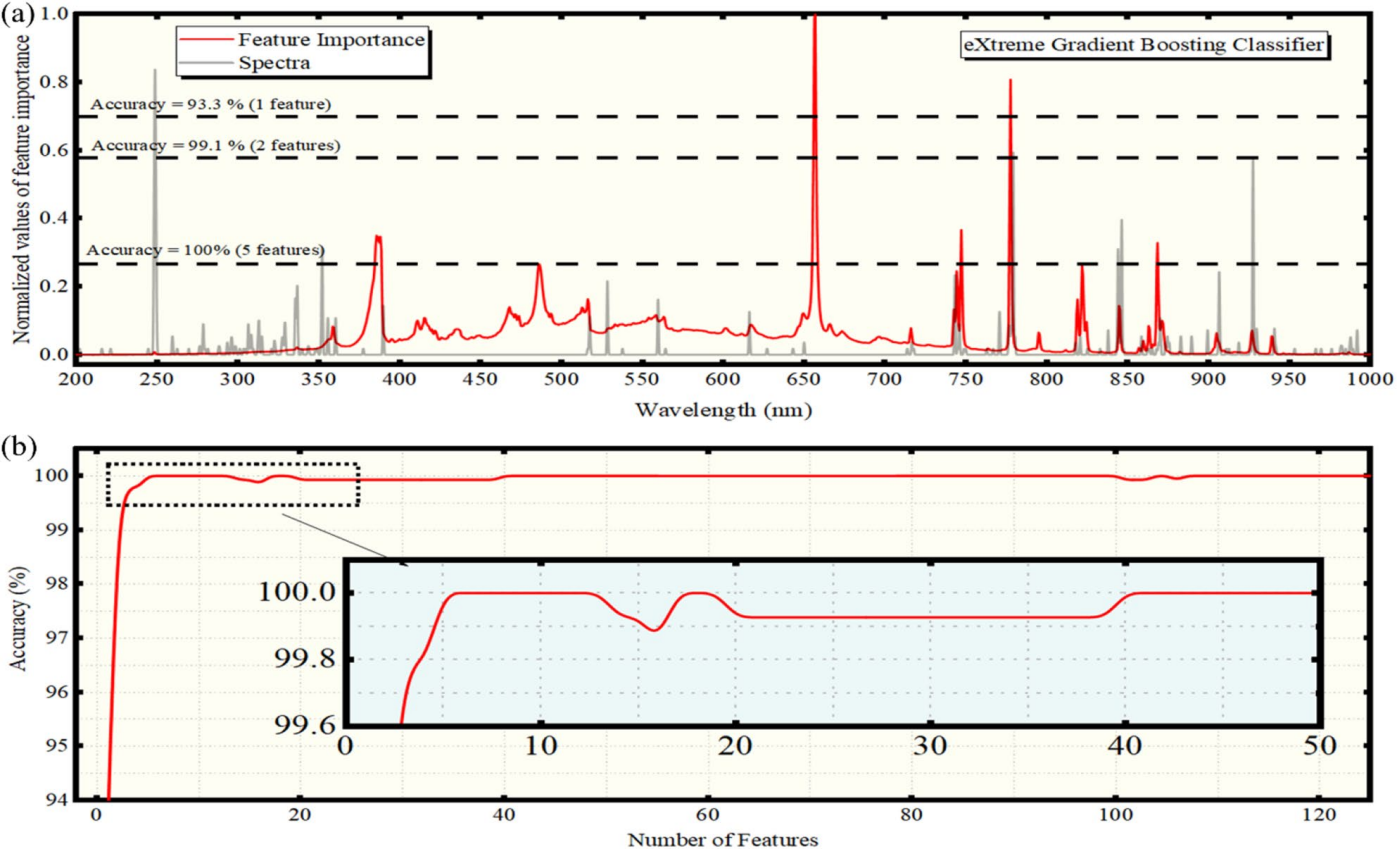
(**a**) Feature importances (gray line) versus wavelength resulting from the XGBoost algorithm. An experimental LIBS spectrum (red line) is shown for comparison. The horizontal dashed lines indicate the number of features threshold and the corresponding computed accuracies. (**b**) Prediction accuracy using the XGBoost algorithm versus the number of features.

Then, the importance of the spectral features present in the LIBS spectra along with the accuracy of the XGBoost algorithm per feature were calculated and are shown in Fig. 4a and b, respectively. As shown in Fig. 4a, when only the C I 247.9 nm emission is used by the algorithm, an accuracy of 93.3% is achieved. However, when the O I 777.5 nm emission is included for the training procedure, an accuracy of 99.1% is attained. The addition of the O I 844.6 nm and 926.4 nm emissions, along with the CN (Δν = − 1) band, in the predictive model, result to further improvement of the accuracy of the algorithm, attaining 100%. It is worth to note that the algorithm attributes some non-negligible importance to some weaker spectral features, such as the NH 336.3 nm and C I 529.7, 906.2 and 940.6 nm emissions, the O I 615.7 nm and 777.2 nm, the N I 744.2, 746.8 and 868.3 nm emissions, and the emissions of the C_2_ (Δν = 0, -1) bands at 516.5 and 563.5 nm. Very interestingly, the contribution of the continuum background is almost eliminated using this algorithm, and practically the spectral lines’ emissions are mainly contributing in the discrimination procedure.

It must be emphasized here that the algorithms selected to be used in the present work are among the most well-known and popular ones for classification purposes. I addition, all of them, with the exception of the LDA, provide the possibility to calculate the feature importances and their relative contribution in the classification, thus allowing the experimentalist to tune the experimental conditions in order to optimize particular emissions with increased weight for the classification. From the comparison of the obtained results, it results that the XGBoost classifier, which is considered among the most powerful machine learning algorithms, and is widely used in a variety of classification tasks, can achieve accuracies similar or even higher than that obtained by the RFC and ERTC algorithms, using considerably less spectral features as well. It is interesting, that using the ERTC algorithm, the background continuum was found to contribute significantly to the classification procedure (see e.g. Fig. 2a). However, even in this case, where the continuum has some importance, the use of C, O and N emissions, results to predictive models capable for discrimination of the olive oil samples with accuracies exceeding 95%. In any case, the continuum contribution was found to be less important in the case of the RFC algorithm (see e.g. Fig. 3a), while, in the case of XGBoost is almost eliminated (see e.g. Fig. 3a), as only two spectral features of the LIBS spectra have been shown to ensure a very successful classification of the olive oil samples.

## Discussion

As mentioned in the Introduction, there are several approaches, based on different analytical techniques assisted by different statistical approaches, that have been proposed for the assessment of the geographical origin of olive oils. NMR, ICP-MS and δ^13^C isotope ratio (Isotope Ratio Mass Spectrometry, IRMSICP), HPLC (e.g., coupled to Charged Aerosol Detector (CAD) and High Temperature-GC-F) and Selected Ion Flow Tube Mass Spectrometry (SIFT-MS) are among those reported recently for such tasks, assisted by statistical analysis methods such as Hierarchical Cluster Analysis (HCA), Principal Component Analysis (PCA), PLS-DA, Soft Independent Modelling by Class Analogy (SIMCA), etc.^22^. In most of the cases, high classification accuracies, better than 90% have been reported. A presentation of the techniques used can be found in a recent report by Cecchi, L. et al.^22^. Very recently, after the initial submission of the present work, some works have appeared in the literature concerning the geographical discrimination of EVOOs, using squalene, tocopherol, and fatty acid composition measurements by chromatographic techniques^46^, optical spectroscopies^47^ and isotopic traceability (^13^C and ^18^O)^48^. All these works report high classification accuracies. However, the degree of the experimental complexity of most of these analytical techniques, and/or the operational and apparatus cost, seem to limit their involvement in fast and routine and/or online operation. In that view, LIBS technique aided by appropriate machine learning algorithms, is more suitable for fast routine, on-line and in-situ operation.

The present results suggest that LIBS technique can provide the necessary spectroscopic information (i.e., the different emissions of the laser induced plasma), for the successful discrimination/classification of olive oils originating from different geographical areas. In order to accomplish this task, and because of the high similarity of the LIBS spectra of the different olive oil samples, LIBS is assisted by different machine learning algorithms. The implementation of the machine learning algorithms enabled the detection of the subtle spectral differences, the identification of spectral motives and/or patterns, and allowed to perform successfully the correlations among the observed emissions and the geographical origin to accomplish the classification. For the analysis of the LIBS spectroscopic data some widely used and powerful machine learning algorithms have been used such as LDA, ERTC, RFC and XGBoost. The selection of the last three algorithms was based on their ability to provide information about the relative contribution (feature importance) of each spectral line of the LIBS spectra on the classification results, thus allowing to shed some more light on the “black-box” operation of most of the machine learning approaches and identify the spectral features with the larger contribution on the classification accuracy. In this way, the physical content of the LIBS spectra can be more straightforwardly correlated with the classification, the whole procedure of statistical analysis acquiring more physical significance and becoming more useful for the spectroscopic community. In addition, this procedure reduces significantly the number of spectral features employed by the algorithms, keeping only the most contributing ones, instead of introducing the entire spectra. This is also important as it can reduce the computation time considerably, while it allows for the tuning of the experimental conditions in favor of the specific spectral features (intensity, resolution, etc.), to maximize the classification accuracy. In the present investigation, this procedure demonstrated that the spectral features (i.e., emissions) which contribute the most for the discrimination, are the emission lines of elements such as carbon, oxygen and nitrogen.

In addition to the above, the present work showed that both the linear (i.e., LDA) and the non-linear (i.e., ERTC, RFC and XGBoost) algorithms can operate successfully and efficiently regarding the geographic classification of olive oil samples using the LIBS spectroscopic data. This finding is of great interest, because it confirms that a variety of machine learning models can be used for such discrimination/classification tasks not limiting the choice to only linear or non-linear algorithms. In all cases, the success of the followed procedure was assessed by performing an internal validation of each predictive model with cross validation, confirming the successful classification achieved.

To further validate the classification results an external validation procedure was applied for all the algorithms used. This was achieved by removing a part of the LIBS data used for the prediction model. Although, all employed algorithms were found operating successfully, attaining very high accuracies, the most successful model was found to be the XGBoost one, since it succeeded in both reducing the initial dataset by thousands of times, retaining only 2 spectral features, while maintaining a very high accuracy up to 99%. This finding can be of great importance in the cases where much larger number of samples have to be treaded and/or when higher resolution spectroscopic instrumentation is used, which can provide larger spectroscopic data sets (i.e., spectral data). For both cases, the datasets can be quite large and a technique capable for efficient reduction of their size, together with the requirement for high predictive performance would be mandatory.

In conclusion, the present study, provides an experimental evidence about the great potential of machine learning assisted LIBS as a tool for discrimination/classification purposes of EVOOs and VOOs samples in terms of their geographical origin. The present approach can be employed to assist testing laboratories and can be easily implemented in activities regarding the authenticity of olive oils aiming to avoid fraud and mislabeling of olive oils, thus operating for the benefit of both consumers and producers. Extension and testing of the present approach for olive oils from different olive oil producing countries is among the future scopes of the present research.

## Materials and methods

### The olive oil samples

139 different virgin olive oil (EVOOs and VOOs) samples were studied originating from different regions of Greece collected within a national research program, i.e. the Emblematic Action “The Olive Road”. Among these samples, 49 olive oil samples were originated from Lesvos (L), 36 from Crete (C) and 54 olive oil samples were from Peloponnese (P). More information about the samples and their code names are presented in Table S2 in the Supplementary Material section. The olive oil samples, after their collection, were stored in dark-colored glass bottles and kept at a temperature of 2–4 °C. Prior to the laser measurements, the oil samples were left at room temperature for about four hours.

### LIBS experimental setup

For the experiments, 2 ml of each olive oil sample was placed in small shallow glass dishes (Petri dish). The laser employed was a 5 ns Q-switched Nd: YAG laser (Quanta-Ray INDI, Spectra Physics) operating at each fundamental wavelength at 1064 nm at an energy of about 90 mJ, ensuring a good signal to noise ratio while keeping splashing of the liquid sample minimum. The laser beam was focused by means of a 10 cm focal length lens on the sample free surface to induce the plasma. The radiation of the plasma was collected with a 2-inch diameter quartz lens and was introduced to a quartz fiber bundle coupled to a portable spectrometer (Avantes, AvaSpec-2048-USB2, 75 mm focal length) for spectral analysis. The spectrometer was equipped with a 300 lines/mm diffraction grating, and a 2048 pixels CCD detector. A time delay (t_d_) of 1.28 μs, and a gate width (t_w_) of 1.05 ms were used for the temporal gating of the detector. For each olive oil sample, 10 consecutive laser shots were performed on the same place and were averaged, corresponding to one LIBS measurement. Then, 30 such independent measurements at different places on the surface of the sample were performed to provide a statistically significant basis for treatment and further statistical evaluation. A schematic of a typical LIBS experimental setup is presented in Fig. S3.

### Data analysis

The analysis of the LIBS spectroscopic data was performed using the open-source machine learning Python library Scikit-learn^49^ using different machine learning techniques in order to test their suitability and efficiency to classify such spectroscopic data. Linear Discriminant Analysis (LDA)^50^, Extremely Randomized Trees Classifier (ERTC)^51^, Random Forest Classifier (RFC)^52^ and eXtreme Gradient Boosting Classifier (XGBoost)^53^ were applied. The supervised algorithms (e.g. LDA, ERTC, RFC and XGBoost) were used for classification purposes, and in the case of LDA, for supervised visualization of the multidimensional spectroscopic data in a lower dimensions space, as well. Furthermore, ERTC, RFC and XGBoost are tree-based methods that have the possibility to select the most important spectroscopic features (e.g. wavelengths) which affect the discrimination between the samples^54,55^. Their main differences are due to the way decision trees are used to predict. ERTC, RFC and XGBoost are all composed by a large number of decision trees and the result is calculated by taking into account the prediction of each tree. However, ERTC uses the whole dataset, while RFC uses bootstrap replicas of the dataset. Moreover, ERTC randomly chooses the way the trees’ nodes are split, while RFC chooses the optimum ones. In that view, the ERTC algorithm is computationally faster than the RFC one but can be more sensitive to noisy features. On the other hand, XGBoost is sequentially and iteratively using trees to compute the optimal nodes, in such a way that trees are learning from previous iterations.

In order to estimate the accuracy and the performance of the predictive models, both internal and external validations were performed. For the former, the k-fold cross-validation technique was used, while for the latter, 4 samples from each region (12 samples in total) were removed from the training procedure and were used only for testing by each predictive model. For a more detailed picture of the algorithmic training, the classification reports and the confusion matrices were also constructed. The former contains the values of merit that are related to the classification, as for instance the precision, the recall, the f1-score values, the support and the weighted average, while the latter represent a performance measurement for machine learning classification and basically presents the correctly and falsely predictions of every sample in details. A more detailed explanation about these metrics and their significance can be found in Ref.^34^.

It should be added at this point, that an extensive grid search for the selection of the optimum algorithmic parameters was not necessary, for any of the algorithms, since with the default values the obtained accuracies were already extremely high. In more details, for LDA algorithm, the maximum number of components (i.e., canonical variables) used for the analysis was two (2). For the other models (i.e., ERTC, RFC, and XGBoost) the default values of the different parameters used can be found in Scikit-learn library^49^. Specifically, the most important parameter for ERTC, RFC and XGBoost algorithms is the number of estimators, which was kept steady at 100 for comparison purposes between the three algorithms, since the value of this parameter affects directly the computed importances.

## Supplementary Information

Supplementary Information
