# Supplementary material for: Laser-induced breakdown spectroscopy coupled with machine learning as a tool for olive oil authenticity and geographic discrimination
Source: Sci Rep. 2021 Mar 8;11:5360. doi: 10.1038/s41598-021-84941-z (PMC7970888; doi:10.1038/s41598-021-84941-z)
Supplement: Supplementary file 1 — Supplementary Information [file 41598_2021_84941_MOESM1_ESM.pdf]

Laser-Induced Breakdown Spectroscopy coupled with machine learning as a tool for olive oil authenticity and geographic discrimination

Nikolaos Gyftokostas[1, 2, ⸸], Dimitrios Stefas[1, 2, ⸸] Vasileios Kokkinos[3, ⸸], Christos Bouras[3] and Stelios Couris[1, 2, *]

[1]Department of Physics, University of Patras, 26504 Patras, Greece

[2]Institute of Chemical Engineering Sciences (ICE-HT), Foundation for Research and Technology-Hellas (FORTH), Patras, Greece

[*]couris@upatras.gr , couris@iceht.forth.gr

[3]Department of Computer Engineering & Informatics University of Patras, 26504 Patras, Greece

[⸸]these authors contributed equally to this work

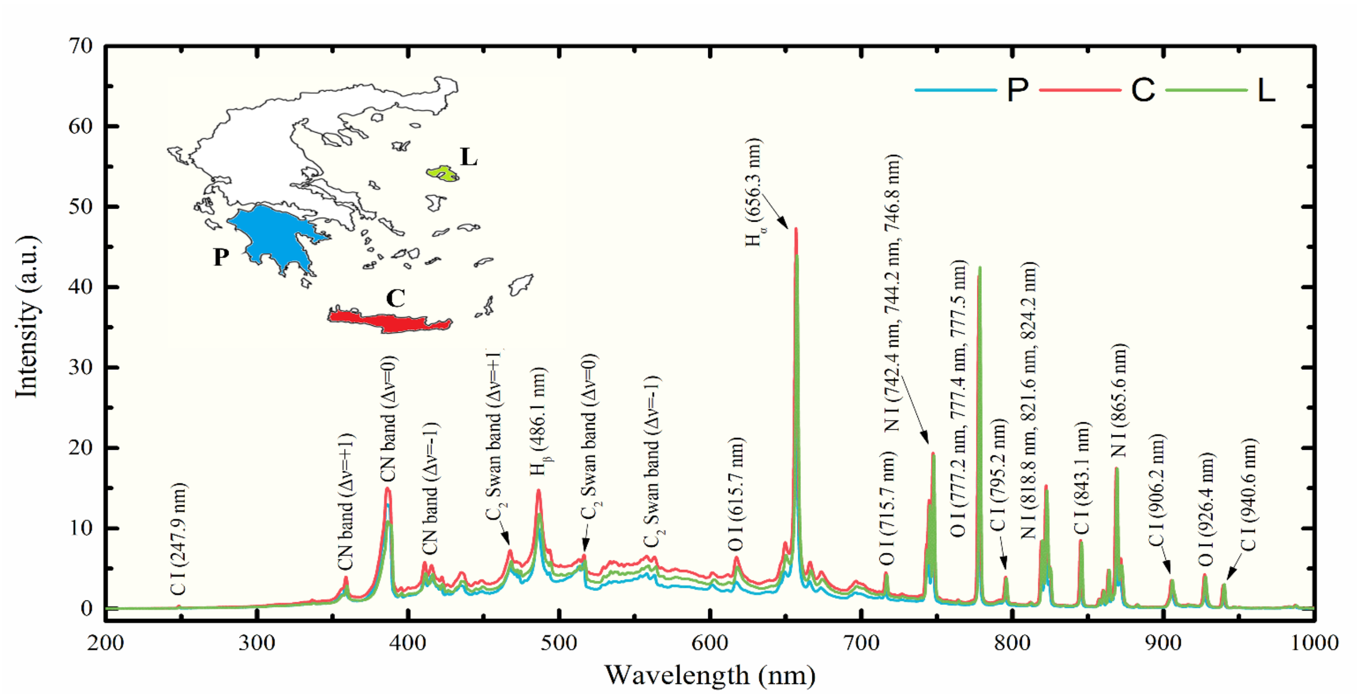

Figure S1. LIBS spectrum of three randomly selected olive oil samples originating from Peloponnese, Crete, and Lesvos, respectively.

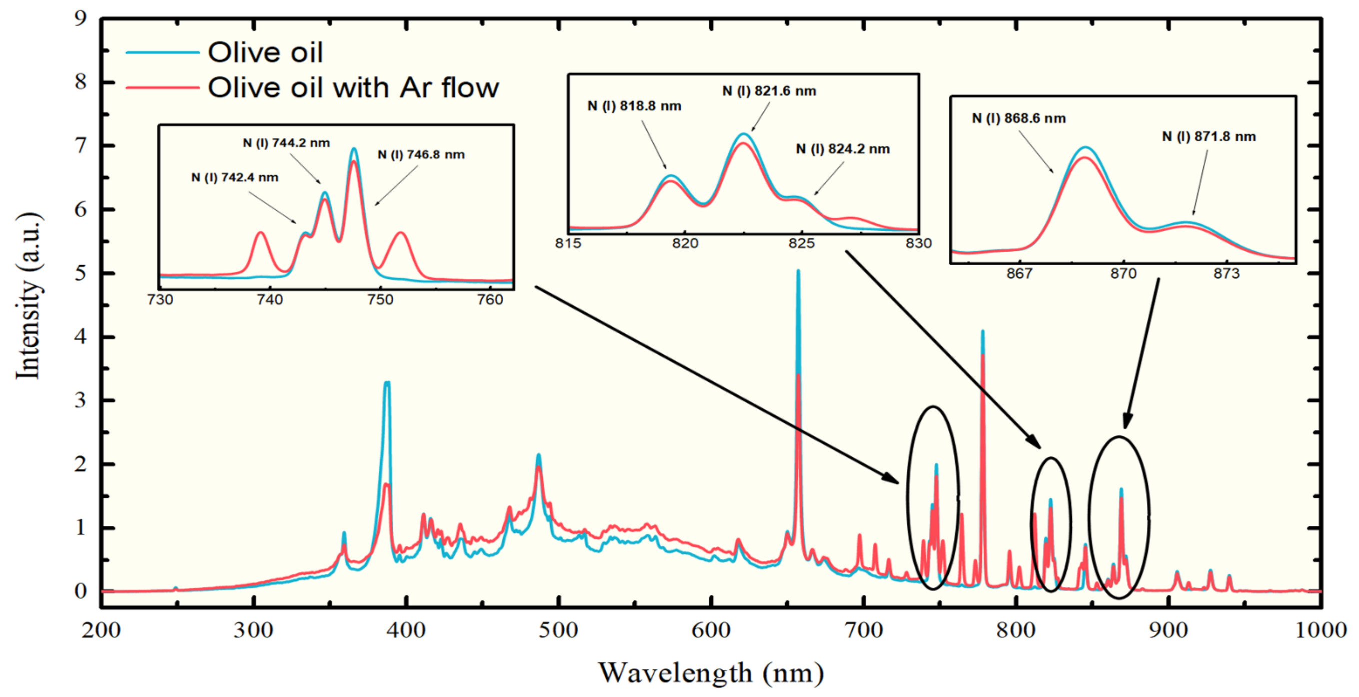

Figure S2. LIBS spectra of an EVOO sample obtained without flowing (blue line) and under flowing (red line) Argon (Ar) gas at the surface of the sample.

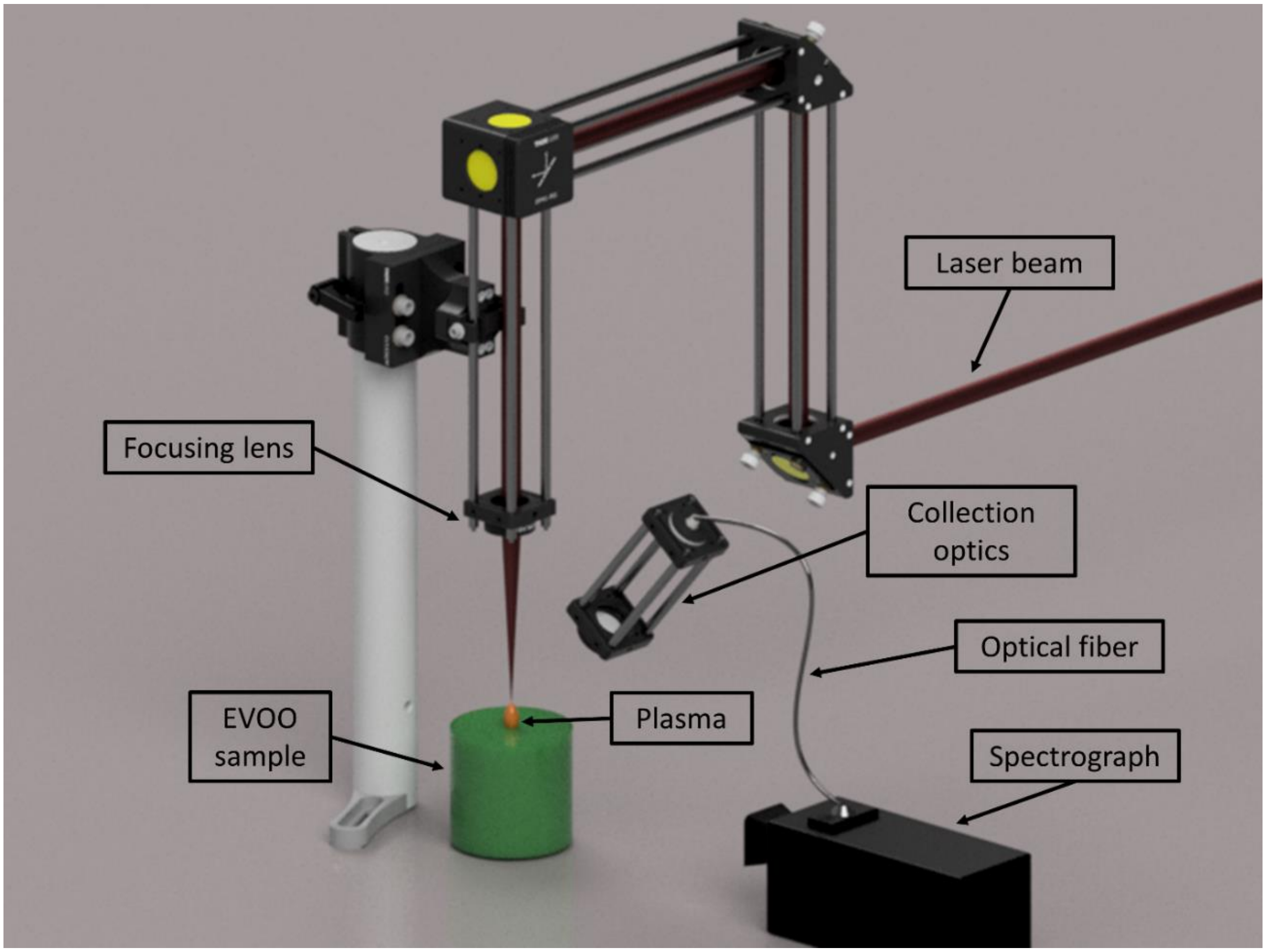

Figure S3. Schematic representation of LIBS experimental setup

Table S1. Observed spectral lines appearing in the olive oil LIBS spectra

**Species***	**Wavelength (nm)**
C I	247.9, 795.2, 833.5, 906.2, 940.6
H I	H_α 656.3, H_β 486.1, H_γ 434.1
N I	410.3, 410.9, 672.6, 694.6, 698.1, 742.4, 744.2, 746.8, 818.8, 821.6, 824.2, 868.3
O I	615.7, 715.7, 777.2, 777.4, 777.5, 844.6, 926.4
CN	350 nm to 430 nm, indicative band heads are (0-0) 388.3, (1-0) 350 and (0-1) 421.6
C_2	450 nm to 570 nm, indicative band heads are (0-0) 516.5, (1-0) 473.7 and (0-1) 563.5
NH	320 nm to 350 nm, indicative band heads are (0-0) 336.3 and (1-1) 337.4

* C I, H I, N I, O I : neutral Carbon, Hydrogen, Nitrogen and Oxygen atomic spectral lines

Table S2. EVOOs and VOOs samples and their code names

Origin		Code Name
Crete	7 from Lasithi	C
	12 from Heraklion	
	8 from Rethymnon	
	9 from Chania	
Peloponnese	3 from Argolida	P
	1 from Filiatra	
	2 from Kalamata	
	5 from Corinth	
	7 from Lakonia	
	36 from Messinia	
Lesvos	49 from Lesvos	L
	Total: 139 olive oil samples	
